# The Role of Mycobacterium indicus pranii in Sepsis Management: A Comprehensive Review of Clinical Outcomes and Therapeutic Potential

**DOI:** 10.7759/cureus.66772

**Published:** 2024-08-13

**Authors:** Devshree Dhande, Archana Dhok, Ashish Anjankar, Shailesh Nagpure, Roshani Ganjare

**Affiliations:** 1 Biochemistry, Jawaharlal Nehru Medical College, Datta Meghe Institute of Higher Education and Research, Wardha, IND; 2 Pharmacology, Dr. Rajendra Gode Medical College, Amravati, IND

**Keywords:** clinical outcomes, therapeutic potential, inflammation, immunomodulation, mycobacterium indicus pranii, sepsis

## Abstract

Sepsis is a critical condition characterized by a dysregulated immune response to infection, leading to systemic inflammation, multi-organ failure, and high mortality rates. Current treatments primarily involve antibiotics and supportive care, which address the infection and stabilize hemodynamics but do not directly modulate the inflammatory response. This limitation highlights the need for novel therapeutic approaches. This review aims to evaluate the role of *Mycobacterium indicus pranii* (MIP) in sepsis management, focusing on its clinical outcomes and therapeutic potential. By examining preclinical and clinical evidence, we seek to understand the efficacy, safety, and practical applications of MIP in treating sepsis.

A comprehensive review of existing literature was conducted, including preclinical studies, clinical trials, and case reports involving MIP. The review synthesizes findings related to its mechanism of action, therapeutic efficacy, and safety profile. MIP has demonstrated significant immunomodulatory effects, including enhancing innate and adaptive immune responses and reducing excessive inflammation. Clinical trials have shown promising results, with MIP improving clinical outcomes and reducing sepsis-related complications. The agent's unique ability to modulate the cytokine storm associated with sepsis positions it as a potential adjunctive therapy. MIP offers a novel approach to managing sepsis by addressing immune dysregulation and inflammation. The evidence suggests that MIP could be a valuable adjunct to current treatments, improving patient outcomes and addressing some limitations of conventional therapies. Further research is needed to establish its role in clinical practice and to optimize treatment protocols.

## Introduction and background

Sepsis is a severe and often life-threatening condition that arises from a dysregulated host response to infection [1]. Characterized by widespread inflammation and systemic organ dysfunction, sepsis typically begins with a microbial infection, which may be bacterial, viral, or fungal. This infection triggers a cascade of inflammatory responses, releasing pro-inflammatory cytokines and mediators [2]. These substances cause systemic inflammation, resulting in endothelial dysfunction, increased vascular permeability, and microvascular thrombosis. The progression of sepsis to severe forms, such as septic shock, is marked by significant cardiovascular instability and multi-organ failure.

The complex pathophysiology of sepsis underscores the need for effective management strategies to address the infection and the resultant inflammatory response [3]. Current sepsis treatments primarily involve administering broad-spectrum antibiotics to combat the causative pathogens alongside supportive care measures such as fluid resuscitation and vasopressor therapy to stabilize hemodynamics. While these approaches are foundational, they have notable limitations [4]. The rise of antibiotic-resistant pathogens and variability in patient responses complicate treatment efficacy. Moreover, supportive measures alleviate symptoms and stabilize patients but do not directly address the underlying inflammatory dysregulation. Consequently, there is an urgent need for adjunctive therapies that can effectively modulate the immune response and improve patient outcomes in sepsis [5].

*Mycobacterium indicus pranii* (MIP), a non-pathogenic soil-dwelling mycobacterium, has emerged as a potential therapeutic agent due to its immunomodulatory properties. Originally isolated from soil, MIP has been studied for its ability to enhance immune responses and modulate inflammation [6]. Historically, MIP has been used in vaccine development and as an adjuvant in treating leprosy and tuberculosis. Its role in these contexts highlights its potential to boost immune function and address infections [7]. In recent years, research has expanded to explore MIP's application in managing sepsis and other inflammatory conditions. Clinical trials have investigated its effects in reducing infection-related complications and improving clinical outcomes.

The therapeutic potential of MIP is attributed to its ability to modulate both innate and adaptive immune responses [8]. It enhances the activation of macrophages and promotes the production of cytokines that mediate immune responses. Additionally, MIP can reduce excessive inflammation by modulating the cytokine storm that often accompanies sepsis. This unique combination of immune enhancement and inflammation control makes MIP a promising candidate for adjunctive therapy in sepsis [9]. This review aims to comprehensively evaluate MIP's role in sepsis management, focusing on its clinical outcomes and therapeutic potential. The objectives include synthesizing evidence from preclinical and clinical studies to provide a thorough understanding of MIP’s efficacy, safety, and practical applications in sepsis treatment. Additionally, the review will compare MIP with existing therapies and explore its potential integration into current sepsis management protocols.

## Review

Mechanism of action

Immune Modulation

MIP significantly influences immune modulation, affecting innate and adaptive immune responses. It induces a Th1 and Th17 immune response while downregulating the Th2 pathway, crucial for enhancing the body's infection response and effectively modulating inflammation. MIP stimulates the activation of macrophages and dendritic cells, essential components of the innate immune response [10]. These cells are vital for pathogen recognition and initiating the immune response. Regarding adaptive immunity, MIP promotes the activation of T cells, particularly the differentiation into Th1 and Th17 cells, which are essential for a robust cell-mediated immune response and the establishment of immunological memory [10]. MIP's interaction with cytokine networks is key to its immune modulation. It enhances the production of pro-inflammatory cytokines, which are critical for the activation and proliferation of immune cells. Specifically, MIP promotes the secretion of key cytokines such as interferon-gamma (IFN-γ) and interleukin-17 (IL-17), which are vital for Th1 and Th17 responses, respectively [11].

These cytokines facilitate the recruitment and activation of other immune cells, including CD8+ T cells and natural killer (NK) cells, amplifying the immune response. Additionally, MIP influences the expression of various chemokines essential for the migration and positioning of immune cells at sites of infection or inflammation, orchestrating a coordinated immune response and enhancing overall immune system effectiveness [11]. The therapeutic potential of MIP lies in its ability to induce protective immune responses, which is crucial for preventing infections and diseases. By promoting a robust Th1 and Th17 response, MIP helps establish long-lasting immunological memory, which is particularly beneficial in preventing recurrent infections and enhancing vaccine efficacy against various pathogens [12]. Moreover, MIP has demonstrated efficacy in various clinical settings, including its use in sepsis, which enhances the immune response against Gram-negative bacteria. Its ability to stimulate both humoral and cell-mediated immunity positions it as a valuable therapeutic agent in infectious diseases and potentially in cancer immunotherapy [12].

Microbial Effects

MIP has demonstrated significant potential in modulating pathogen load, especially in sepsis. The immunomodulatory properties of MIP enhance the host's immune response, leading to a substantial reduction in pathogen load [8]. Research indicates that MIP stimulates a Th1-type immune response, crucial for combating infections and controlling bacterial proliferation during septic episodes. Clinical studies involving sepsis patients have shown that MIP treatment is associated with decreased secondary infections, such as ventilator-associated pneumonia (VAP) and catheter-related bloodstream infections (CRBSI). These findings suggest that MIP not only enhances immune function but also plays a role in reducing the overall pathogen load in critically ill patients [8].

Beyond its effects on direct pathogen interactions, MIP also influences the modification of microbial flora within the host. During sepsis, the balance of microbial communities, particularly in the gut, is often disrupted, leading to dysbiosis, which can exacerbate immune dysfunction and increase susceptibility to infections. MIP's ability to enhance the immune response may help restore a healthier balance of microbial communities [13]. MIP improves clinical outcomes by promoting an environment conducive to beneficial microbes while inhibiting pathogenic species. A balanced microbiome is essential for maintaining immune homeostasis and preventing opportunistic infections, further underscoring the therapeutic potential of MIP in sepsis management [13].

Anti-Inflammatory Effects

MIP exhibits significant anti-inflammatory effects through several key mechanisms that help reduce excessive inflammation. One primary way MIP accomplishes this is by modulating cytokine production. It promotes a shift towards a Th1 immune response, characterized by the secretion of pro-inflammatory cytokines such as interferon-gamma (IFN-γ) [14]. This shift is crucial as it helps balance the immune response by downregulating Th2 cytokines, which can exacerbate inflammation. By favoring a Th1 response, MIP enhances the body’s ability to combat infections while controlling inflammation [14]. In addition to cytokine modulation, MIP inhibits the activity of macrophage inflammatory proteins (MIPs), such as MIP-1α and MIP-2.

These chemokines significantly mediate acute inflammatory responses by recruiting and activating neutrophils [15]. By suppressing the production and activity of these chemokines, MIP can reduce neutrophil recruitment, mitigating excessive inflammation and preventing tissue damage associated with an overactive inflammatory response. Furthermore, MIP may inhibit Toll-like receptor (TLR) signaling pathways, which are crucial for recognizing pathogens and initiating inflammatory responses. By blocking TLR-mediated pathways, MIP decreases the production of inflammatory mediators, reducing tissue damage and inflammation [15].

MIP offers distinct advantages over conventional anti-inflammatory treatments, such as non-steroidal anti-inflammatory drugs (NSAIDs) and corticosteroids. NSAIDs primarily work by inhibiting cyclooxygenase (COX) enzymes to reduce the synthesis of prostaglandins, while corticosteroids broadly suppress immune responses. In contrast, MIP specifically modulates immune pathways and cytokine production, providing a more targeted approach to managing inflammation [16]. Additionally, MIP has a favorable safety profile as a non-pathogenic mycobacterium, presenting a potentially safer alternative to long-term corticosteroid use, which can lead to significant side effects, including immunosuppression and an increased risk of infections [16].

**Figure 1:**
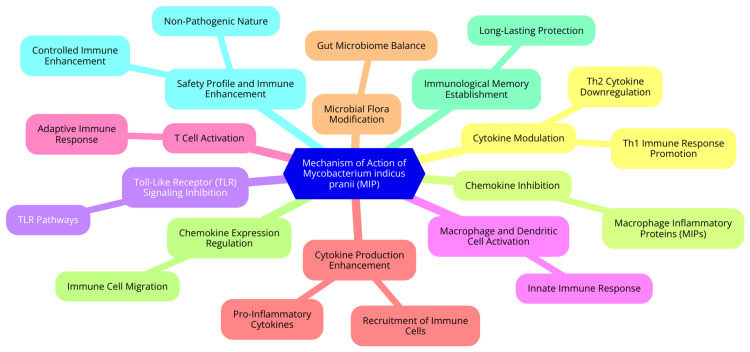
Mycobacterium indicus pranii (MIP): the mechanism of action Image Credit: Dr. Renuka Potond

Another notable advantage of MIP is its potential for immune enhancement. Unlike traditional anti-inflammatory agents that may suppress the immune system, MIP can enhance immune responses in a controlled manner [17]. This characteristic is particularly beneficial in compromised immune function, such as sepsis. Overall, MIP's ability to modulate cytokine and chemokine activity, inhibit TLR signaling, and regulate neutrophil responses positions it as a promising candidate for anti-inflammatory therapy. Further research is necessary to fully elucidate its therapeutic potential and establish its efficacy compared to existing treatments [17]. The mechanism of action of MIP is shown in Figure 1.

Clinical evidence

Preclinical Studies

MIP has shown significant promise in preclinical studies, particularly in animal models of sepsis. These studies have employed various methodologies to assess MIP's efficacy and potential role as an immunotherapeutic agent in managing sepsis [8]. Animal models, especially guinea pigs and mice, have been crucial in evaluating MIP's effects. In guinea pig models, MIP has demonstrated higher immunogenicity than the Bacillus Calmette-Guérin (BCG) vaccine, which is traditionally used for tuberculosis vaccination. This model allows researchers to assess MIP's immune response and protective efficacy against infections [18]. Mouse models, on the other hand, have been utilized to explore both prophylactic and therapeutic applications of MIP. In these studies, animals were immunized with heat-killed MIP and subsequently exposed to pathogens or conditions that induce sepsis, enabling researchers to evaluate the protective effects of MIP under controlled circumstances [18].

Key methodologies in these preclinical studies included various immunization protocols, where animals received doses of heat-killed MIP followed by exposure to sepsis-inducing agents. Researchers measured immune responses by analyzing cytokine profiles, particularly focusing on producing Th1-type cytokines essential for a robust immune response. Clinical outcomes such as survival rates, bacterial load, and organ function were closely monitored post-treatment. The results indicated that MIP treatment significantly reduced mortality rates in septic mice and improved organ function, suggesting its potential as an adjunct therapy in sepsis management [19].

The findings from these preclinical studies highlight several important aspects of MIP's efficacy. Firstly, MIP has shown enhanced immunogenicity compared to BCG, indicating that it may serve as a more effective immunotherapeutic agent. Secondly, treatment with MIP significantly reduced mortality rates associated with sepsis, underscoring its therapeutic potential [20]. Furthermore, MIP administration was associated with a favorable shift in the immune response, characterized by increased production of Th1 cytokines, crucial for combating infections during sepsis. Finally, studies suggest that MIP not only aids in managing sepsis but also protects against secondary infections, a common complication in septic patients [20].

Clinical Trials and Case Studies

Recent clinical trials involving MIP have provided valuable insights into its potential role in sepsis management. One key aspect of these trials is their design, emphasizing the need for robust methodologies to assess treatment efficacy accurately. For instance, a comprehensive approach to sepsis trials has been advocated, focusing on animal models to understand sepsis pathophysiology better. This approach aims to identify specific biomarkers and molecular signals to categorize patients more effectively, allowing for tailored treatments. Additionally, the importance of developing networks of experienced investigators and employing adaptive trial designs has been underscored, as these strategies can enhance the quality and relevance of the findings [8].

A systematic review of clinical trials in sepsis has also highlighted the challenges faced in past studies. Many trials have struggled to demonstrate significant benefits in mortality rates, indicating a need for improved measures of illness severity and better identification of high-risk patients. The review suggests that future trials should consider novel endpoints beyond all-cause mortality, such as organ dysfunction and specific treatment responses. This shift in focus could provide more informative outcomes and ultimately lead to more effective interventions for sepsis patients [21].

In addition to clinical trials, case studies have illustrated the practical application of MIP in sepsis management. One notable example comes from Baptist Health South Florida, where a concerted effort to improve sepsis care led to remarkable outcomes. The institution reported a significant decrease in the mortality rate for sepsis patients, dropping from 1.91% in early 2017 to just 0.45% by 2019. This improvement was accompanied by increased compliance with treatment bundles, which rose from 52% to 88% during the same period. Furthermore, the average length of stay for sepsis patients was reduced from 6.83 days to 3.88 days, highlighting the effectiveness of the implemented strategies [8].

The success at Baptist Health South Florida can be attributed to the establishment of a virtual sepsis unit and the standardization of care processes, which enhanced compliance with evidence-based guidelines. This case study is a compelling example of how clinical experience and structured protocols can improve patient outcomes in sepsis management. Collectively, these trials and case studies underscore the potential of MIP and other innovative approaches in addressing the complexities of sepsis, paving the way for more effective treatment strategies in the future [22].

Safety and Tolerability

MIP has been investigated for its safety and tolerability, particularly in therapeutic contexts such as sepsis management. Overall, MIP has demonstrated a favorable safety profile, with most adverse effects reported being mild to moderate [10]. Common adverse effects include local reactions at the injection site, such as pain, swelling, and redness, which typically resolve quickly. Additionally, some patients may experience systemic reactions resembling flu-like symptoms, including fever, malaise, and fatigue shortly after administration. There have also been mild hematological changes, such as transient thrombocytopenia, anemia, and leukopenia, though these effects generally resolve without requiring clinical intervention [10].

Serious adverse events associated with MIP are rare. Clinical studies have reported no treatment-related deaths or severe adverse events necessitating the discontinuation of therapy, indicating that MIP can be administered safely in appropriate clinical settings. This is particularly important in the context of sepsis, where patients are often vulnerable and may have compromised immune systems. The low incidence of serious adverse effects reinforces the potential of MIP as a therapeutic option for managing sepsis [23].

Long-term safety considerations are also crucial when evaluating MIP's overall profile. While long-term studies focused on MIP are limited, the available data suggest that it does not lead to significant long-term adverse effects. Monitoring patients receiving MIP therapy is advisable to identify any delayed reactions or complications [24]. Additionally, given MIP's role in modulating immune responses, there is a potential concern regarding its long-term impact on immune function. However, current evidence indicates that MIP does not induce immunosuppression, which is particularly relevant for patients at risk of infections due to their underlying conditions [24].

Despite the promising findings regarding the safety and tolerability of MIP, further research is essential to understand its long-term safety profile fully. More extensive studies are needed to evaluate its effects over prolonged periods and in diverse patient populations, especially those with pre-existing health conditions or those undergoing concurrent immunosuppressive therapies. In summary, MIP appears to have a favorable safety and tolerability profile, with most adverse effects being mild and manageable, making it a promising candidate for further exploration in therapeutic applications [25].

Comparison with existing therapies

Current Sepsis Treatments

Sepsis management involves a multifaceted approach that includes antibiotics, supportive care, and immunomodulatory agents. Each component is crucial in improving outcomes for patients with this complex condition [26]. Antibiotics are critical in treating sepsis and should be initiated immediately, ideally within the first hour of recognition. Broad-spectrum antibiotics are typically used initially to cover a wide range of potential pathogens, especially since definitive identification of the causative organism may take time. Once specific pathogens are identified through cultures, antibiotic therapy may be adjusted to target the identified bacteria more precisely. This tailored approach is crucial to avoid the adverse effects of broad-spectrum antibiotics and combat antibiotic resistance. However, the increasing prevalence of multidrug-resistant organisms complicates treatment, necessitating careful selection and stewardship to optimize antibiotic use and minimize resistance development [27].

Supportive care is essential for restoring hemodynamic stability in septic patients. Intravenous fluid resuscitation is a cornerstone of treatment, particularly in cases of septic shock. Early goal-directed fluid therapy has been shown to improve survival rates by ensuring adequate tissue perfusion [28]. If blood pressure remains low despite adequate fluid resuscitation, vasopressors are administered to constrict blood vessels and elevate blood pressure, with norepinephrine being the most commonly used agent. In addition to fluids and vasopressors, patients may require further interventions such as oxygen therapy, mechanical ventilation, or dialysis if organ dysfunction occurs. Surgical intervention may also be necessary to remove sources of infection, such as abscesses or infected tissues, further underscoring the complexity of sepsis management [28].

Immunomodulatory agents aim to correct the dysregulated immune response seen in sepsis. While traditional treatments focus on infection control and hemodynamic stabilization, these agents enhance the host's immune response to better manage the infection and prevent organ failure [29]. Various immunomodulatory therapies are under investigation, including cytokine inhibitors and other novel agents. However, many of these therapies still need to demonstrate consistent benefits in clinical trials, highlighting the need for ongoing research. The potential of immunomodulatory agents to improve outcomes in sepsis remains an exciting avenue for future exploration [29].

Benefits and Limitations of MIP

MIP has emerged as a promising immunomodulatory agent in managing sepsis, offering several potential benefits compared to standard treatments. One of the most significant advantages of MIP is its association with reduced mortality rates. A systematic review indicated that patients treated with MIP experienced a 43% lower mortality rate than control groups, although this finding did not reach statistical significance. Additionally, MIP has been linked to shorter stays in intensive care units (ICUs) and reduced durations of mechanical ventilation. This could be particularly beneficial in managing healthcare resources and improving patient turnover in critical care settings [30].

Another notable benefit of MIP is its ability to lower the incidence of secondary infections, common complications in septic patients. By stimulating a Th1 immune response, MIP may help reverse the immunosuppression often seen in sepsis, enhancing the body’s ability to fight infections. Furthermore, evidence suggests that MIP may facilitate bacterial clearance. In one study, a significantly higher proportion of patients in the MIP group achieved sputum culture conversion in the fourth week compared to those receiving a placebo, highlighting its potential role in improving clinical outcomes [31].

Despite these promising benefits, there are limitations and considerations regarding using MIP in sepsis management. One of the primary concerns is the need for more extensive research. While initial studies have shown positive outcomes, further large-scale randomized controlled trials are essential to conclusively establish the efficacy and safety of MIP in this context. Additionally, the precise mechanisms by which MIP exerts its immunomodulatory effects remain unclear, necessitating further investigation to fully understand its role in sepsis treatment [32]. Moreover, although MIP is generally well-tolerated, it is not without potential adverse effects. Common reactions include local site erythema and swelling, rare fever occurrences, and other systemic reactions. These factors must be weighed against the benefits when considering MIP as part of a sepsis management strategy [33].

Therapeutic potential and future directions

Integration into Clinical Practice

Integrating MIP into clinical practice for managing sepsis involves establishing protocols and addressing potential challenges. Key aspects for incorporation include patient selection, dosage and administration, combination therapy, monitoring and assessment, and participation in clinical trials and research [8]. Patients diagnosed with sepsis within 48 hours of the onset of the first organ dysfunction are potential candidates for MIP treatment. Inclusion criteria may consist of adults aged 18-60, with considerations for specific comorbidities. Based on existing research demonstrating its efficacy in stimulating the Th1 immune response, MIP can be administered intradermally or through other routes.

Standardized dosing protocols should be developed based on current clinical trial data. MIP should be considered an adjunct to standard sepsis treatment protocols, including antibiotics and supportive care, to enhance the immune response while managing the infection [34]. Regular monitoring of clinical outcomes, such as 28-day mortality, ICU length of stay, vasopressor support duration, and incidence of secondary infections, should be implemented. The Sequential Organ Failure Assessment (SOFA) score can be used to evaluate organ function over time. Participation in ongoing clinical trials should be encouraged to gather additional data on MIP's efficacy and safety in sepsis management, which will help refine protocols and establish best practices [35].

However, several challenges may arise during the implementation process. Integrating MIP into clinical practice may require additional regulatory approvals, particularly if it is classified as a new therapeutic agent, which can be time-consuming and complex. Developing standardized protocols for MIP administration across various healthcare settings can be challenging, and variability in practice may lead to inconsistent outcomes [36]. Educating healthcare providers on the use of MIP, its benefits, and potential side effects is crucial, and this training must be thorough to ensure proper implementation and patient safety. Overcoming skepticism from clinicians accustomed to traditional sepsis management protocols requires robust evidence from clinical trials demonstrating MIP's effectiveness. Ensuring the availability of MIP and related resources in healthcare facilities, particularly in low-resource settings, is essential for successful integration [37].

Current research and future directions

Ongoing research into MIP explores its therapeutic potential in managing sepsis. Several clinical trials are currently evaluating the efficacy of MIP as an adjunct therapy for sepsis patients. For example, a retrospective cohort observational study compares the effects of MIP with standard care to assess its impact on mortality rates and recovery times. Other trials are investigating the safety and immunotherapeutic potential of MIP, aiming to reduce mortality, shorten ICU stays, and lower the incidence of secondary infections such as ventilator-associated pneumonia (VAP) and catheter-related bloodstream infections (CRBSI) [38].

Recent systematic reviews and meta-analyses have begun to offer promising evidence regarding MIP's potential in sepsis therapy. A pooled analysis of two randomized controlled trials involving 252 participants suggested that MIP treatment was associated with a 43% reduction in mortality compared to control groups. However, this finding did not achieve statistical significance. Additionally, patients receiving MIP experienced fewer days on mechanical ventilation and shorter ICU stays, indicating that MIP may promote faster recovery from severe infections. Improvements in delta Sequential Organ Failure Assessment (SOFA) scores also suggest enhanced overall organ function during treatment. These findings underscore MIP's potential to effectively modulate the immune response in septic patients [8].

Future research is crucial to fully understand MIP's role in sepsis management. Expanding clinical trials to include larger sample sizes and diverse patient populations, particularly those with drug-resistant infections, will be important. Investigating combination therapies that integrate MIP with other immunomodulatory agents or novel sepsis treatments could enhance its efficacy. Additionally, mechanistic studies will be essential for elucidating the specific immune pathways activated by MIP and understanding how these pathways can help restore the dysregulated immune response characteristic of sepsis [32].

## Conclusions

Based on our findings, MIP is a promising adjunctive therapy for managing sepsis, offering potential benefits beyond standard treatments. Its unique immunomodulatory properties - enhancing immune responses while controlling excessive inflammation - make it an intriguing candidate for addressing the complex challenges associated with sepsis. The review highlights that conventional therapies focus primarily on infection control and supportive care. However, MIP could address the underlying immune dysregulation and inflammatory responses that often complicate sepsis. Although preliminary evidence from preclinical and clinical studies is promising, further research is essential to fully understand its efficacy, safety, and practical applications. Exploring MIP's role in sepsis management could lead to more effective treatment strategies, ultimately improving patient outcomes and advancing our approach to one of the most critical conditions in modern medicine.
